# Interest in Health Behavior Intervention Delivery Modalities Among Cancer Survivors: A Cross-Sectional Study

**DOI:** 10.2196/cancer.5247

**Published:** 2016-02-11

**Authors:** Emily C Martin, Karen Basen-Engquist, Matthew G Cox, Elizabeth J Lyons, Cindy L Carmack, Janice A Blalock, Wendy Demark-Wahnefried

**Affiliations:** ^1^ University of Texas MD Anderson Cancer Center Department of Behavioral Science Houston, TX United States; ^2^ The University of Texas Medical Branch Department of Nutrition and Metabolism Galveston, TX United States; ^3^ University of Texas MD Anderson Cancer Center Department of Palliative, Rehabilitation and Integrative Medicine Houston, TX United States; ^4^ University of Alabama at Birmingham Department of Nutrition Sciences Birmingham, AL United States

**Keywords:** cancer survivor, technology, smartphone, behavioral intervention, physical activity, diet

## Abstract

**Background:**

Effective, broad-reaching channels are important for the delivery of health behavior interventions in order to meet the needs of the growing population of cancer survivors in the United States. New technology presents opportunities to increase the reach of health behavior change interventions and therefore their overall impact. However, evidence suggests that older adults may be slower in their adoption of these technologies than the general population. Survivors’ interest for more traditional channels of delivery (eg, clinic) versus new technology-based channels (eg, smartphones) may depend on a variety of factors, including demographics, current health status, and the behavior requiring intervention.

**Objective:**

The aim of this study was to determine the factors that predict cancer survivors’ interest in new technology-based health behavior intervention modalities versus traditional modalities.

**Methods:**

Surveys were mailed to 1871 survivors of breast, prostate, and colorectal cancer. Participants’ demographics, diet and physical activity behaviors, interest in health behavior interventions, and interest in intervention delivery modalities were collected. Using path analysis, we explored the relationship between four intervention modality variables (ie, clinic, telephone, computer, and smartphone) and potential predictors of modality interest.

**Results:**

In total, 1053 respondents to the survey (56.3% response rate); 847 provided complete data for this analysis. Delivery channel interest was highest for computer-based interventions (236/847, 27.9% very/extremely interested) and lowest for smartphone–based interventions (73/847, 8.6%), with interest in clinic-based (147/847, 17.3%) and telephone-delivered (143/847, 16.9%) falling in between. Use of other technology platforms, such as Web cameras and social networking sites, was positively predictive of interest in technology-based delivery channels. Older survivors were less likely to report interest in smartphone–based diet interventions. Physical activity, fruit and vegetable consumption, weight status, and age moderated relationships between interest in targeted intervention behavior and modality.

**Conclusions:**

This study identified several predictors of survivor interest in various health behavior intervention delivery modalities. Overall, computer-based interventions were found to be most acceptable, while smartphones were the least. Factors related to survivors’ current technology use and health status play a role in their interest for technology-based intervention versus more traditional delivery channels. Future health behavior change research in this population should consider participants’ demographic, clinical, and lifestyle characteristics when selecting a delivery channel. Furthermore, current health behavior interventions for older cancer survivors may be best delivered over the Internet. Smartphone interventions may be feasible in the future following further adoption and familiarization by this particular population.

## Introduction

Currently, there are an estimated 14.5 million cancer survivors in the United States; they comprise approximately 4% of the population [1]. This number has nearly doubled in the past 15 years [2] and is projected to increase by another 4 million over the next decade [1]. This growing number of cancer survivors has brought to the forefront a host of physiological (eg, lymphedema [3], sexual dysfunction [4], and fatigue [5]) and psychological (eg, anxiety [6] and depression [7,8]) sequelae that follow a cancer diagnosis and its treatment. These consequences and other unique health aspects of surviving cancer increase patients’ risk for health conditions [9]. As an example, second primary cancers among survivors account for 16% of all incident cancer diagnoses and can be attributed to a variety of factors, including both treatment-related issues and lifestyle behaviors [10-12]. Despite this heightened risk, the incidence of risky health behaviors remains high in this population. Data collected in 2009 from the Behavioral Risk Factor Surveillance System indicate that 15.1% of cancer survivors are smokers, 27.5% are obese, and 31.5% do not engage in any form of leisure-time physical activity [13]. More recent estimates indicate that over 30% of adult cancer survivors are obese [14]. There is a clear need for effective behavior change interventions for this population.

The ultimate public health impact of any behavior change intervention is influenced by intervention efficacy as well as reach, adoption, implementation, and maintenance [15]. Interventions that use new technologies, such as smartphones or Web-based tools, have greatly increased reach compared to traditional face-to-face interventions [16-18]. Though technology-based interventions have demonstrated efficacy [19-21], they are typically implemented in younger, healthier populations, rather than older adults with cancer. About 60% of cancer survivors are over the age of 65 years [1]—an age group whose adoption of technology is growing but still lags behind that of the general population [22]. Previous research has shown mixed findings for survivor preferences between more traditional delivery modalities (eg, face to face or telephone) versus technology-based platforms (eg, computer or phone), depending on the behavioral target of the intervention (eg, physical activity or diet) and other demographic and health variables [23,24]. For example, Eakin et al found that breast cancer survivors participating in their telephone-based exercise intervention were more likely to report interest in the same intervention being delivered face to face versus the Internet (83% vs 76%) [25]. Age has also been shown to be predictive of delivery preference [26] and use [27,28]. Increasingly, specific information is needed to understand cancer survivors’ preferences for intervention modalities in order to design programs that produce the greatest public health impact. This need is particularly important given the rapid changes in broad-reaching technology today.

The purpose of this study was to investigate cancer survivors’ interest in four health behavior intervention delivery modalities and to identify the factors that are predictive of interest in new technology (ie, interventions delivered via computers or smartphones) and traditional channels of delivery (ie, interventions that are clinic or telephone-based). Specifically, we were interested in whether interest in different health behavior interventions predicted interest in intervention delivery modality while controlling for demographic characteristics and current health behavior status.

## Methods

### Recruitment

The data in this study were collected in 2010 via a cross-sectional survey mailed to 1917 early-stage breast, colorectal, and prostate cancer survivors [29]. All survey recipients were 18 years of age or older and had completed their primary cancer treatment at the University of Texas MD Anderson Cancer Center (Houston, Texas) within the previous 20 years. We employed a stratified sampling plan to assure representation across the cancer continuum and time from diagnosis (ie, 0-6 months, 6-12 months, 1-5 years, and 5+ years). Patients were selected who had no history of other cancers (with the exception of non-melanoma skin cancer), had no evidence of metastatic disease at the time of recruitment, and who were residents of Harris County or adjacent counties in Southeastern Texas. A reminder postcard and up to three follow-up mailings of the survey were mailed to non-respondents.

### Measures

The survey was meant to inform future planning for lifestyle interventions for cancer survivors and included questions about current health behavior practices (eg, diet and physical activity) and the level of interest in lifestyle interventions and delivery preference. Demographic data regarding patients’ education and marital status were collected. Body Mass Index (BMI) was calculated based on participants’ reported height and weight. Participants were also asked yes/no questions regarding their access to and use of computers, social networking sites, and Web cameras. Participants’ daily consumption of fruits and vegetables was assessed using the National Cancer Institute’s Multifactor Screener, which assesses patients’ dietary habits and intake of 16 different food types in the previous 30 days [30]. Physical activity was measured using a 3-item modified version of the Godin Leisure Time Exercise Questionnaire [31]. This measure produces a weekly leisure activity score based on participants’ weekly frequency of strenuous, moderate, and light physical activity. In addition, participants were asked to rank their interest in learning more about certain health behavior topics (ie, exercise, nutrition, and weight control) on a 5-point Likert scale, ranging from “extremely interested” to “not at all interested.” Finally, participants were asked to rate their interest in receiving this information through a number of different modalities, including clinic-based programs (classes), telephone calls with a health counselor, computer-based programs (eg, using the Internet or a Web camera), and smartphones (eg, iPhone). Again, participants were asked to rate their interest on the Likert scale described above.

### Statistical Analyses

All analyses were conducted using Mplus software version 7.2 [32]. Full information maximum likelihood was used to estimate missing data. In addition, *t* tests and chi-square analyses were used to compare demographic information between those with and without missing data. Given the large sample size for this study, path analysis was used to account for correlations between exogenous and endogenous variables [33]. The four intervention modality variables (ie, clinic, telephone, computer, and smartphone) were regressed onto 14 predictors and 13 interaction terms. The predictor variables consisted of interest in different types of interventions (eg, healthy eating, weight control, and exercise), demographic variables, and diet and physical activity. Interaction terms were created by mean-centering the hypothesized lower-order continuous predictors (eg, age, BMI, physical activity, and fruit and vegetable consumption) and multiplying them by each of the four intervention modality variables (ie, clinic, telephone, computer, and smartphone). Standard errors were estimated via bias-corrected bootstrap with 2000 bootstrap samples, which has been shown to increase power and decrease bias due to non-normally distributed outcomes [34].

## Results

A total of 1917 patients were identified for this survey study; of these, 37 had incorrect addresses and nine were deceased. Out of a possible sample of 1871 patients, 1053 responded to the survey, for a response rate of 56.3%. From these responses, 847 were included in the analysis (206 were excluded because they were missing data for categorical exogenous variables, which cannot be estimated by the full maximum likelihood method). Survey respondents’ characteristics are reported in Table 1.

**Table 1:** Participant characteristics.

Demographic characteristic		Participant data (N=847)
**Cancer type, n (%)**		
	Breast	429 (50.7)
	Colorectal	86 (10.2
	Prostate	332 (39.2)
Mean years since diagnosis, mean (SD)		4.6 (3.1)
Age in years, mean (SD)		61.7 (11.1)
Sex, female, n (%)		471 (55.6)
BMI (kg/m^2^), mean (SD)		27.8 (5.5)
Mean daily fruit and vegetable servings, mean (SD)		5.1 (2.0)
**Education, n (%)**		
	Less than 6^th^ grade	14 (1.7)
	6^th^-11^th^ grade	42 (5.0)
	High school graduate	113 (13.3)
	Trade/Tech/Vocational/Some college	204 (24.1)
	College graduate/post grad	474 (56.0)
**Physical activity, median minutes/week**		
	Light	27.5
	Moderate	30
	Strenuous	0
**Technology use and access, n (%)**		
	Own a computer	751 (88.7)
	Access to Internet in home	528 (62.3)
	Use social networking sites	257 (30.3)
	High-speed Internet in home	493 (58.2)
	Use a Web cam	200 (23.6)

The mean age of participants was 61.7 (SD 11.1) years, with 55.6% (471/847) being female. The mean reported time since a patient’s primary cancer diagnosis was 4.6 (SD 3.1) years. The average BMI for this sample was 27.8 (overweight), with an average of 5.1 reported fruit and vegetable servings per day, and a median of 30 reported minutes of moderate physical activity per week. Analyses were conducted to compare basic demographic variables between participants who were excluded because of missing data and included participants. No significant differences were detected for sex, cancer site, years since diagnosis, BMI, fruit and vegetable consumption, and physical activity. However, a significant difference was found for age: the mean age of those with missing data was 67.2 years, and the mean age of those with no missing data was 61.7 years (*P*<.001). Since age was included in the full information maximum likelihood model used to estimate missing data, the results are unlikely to be biased [35].

Survivor interest in intervention types and modalities is presented in Table 2. Most notably, participants’ interest in smartphone-based interventions was the lowest, with 69% “not at all interested,” while computer-based interventions received the highest percentage of “very” and “extremely” interested.

**Table 2:** Percentage of participants interested in intervention types and delivery modalities.

Interest variable		Not at all interested, %	A little interested, %	Somewhat interested, %	Very interested, %	Extremely interested, %
**Intervention type**						
	Getting in shape (exercise)	18.4	14.5	20.7	26.2	20.2
	Eating better to stay healthy	16.2	11.1	18.1	29.4	25.3
	Weight control	22.7	12.2	15.5	25.9	23.8
**Delivery modality**						
	Clinic-based program	48.4	13.8	15.3	9.3	8.0
	Telephone calls with a health counselor	49.2	16.1	12.6	9.6	7.3
	Computer-based program	36.6	10.2	20.7	17.2	10.7
	Smartphone	68.9	6.6	6.8	4.1	4.5

**Figure 1:**
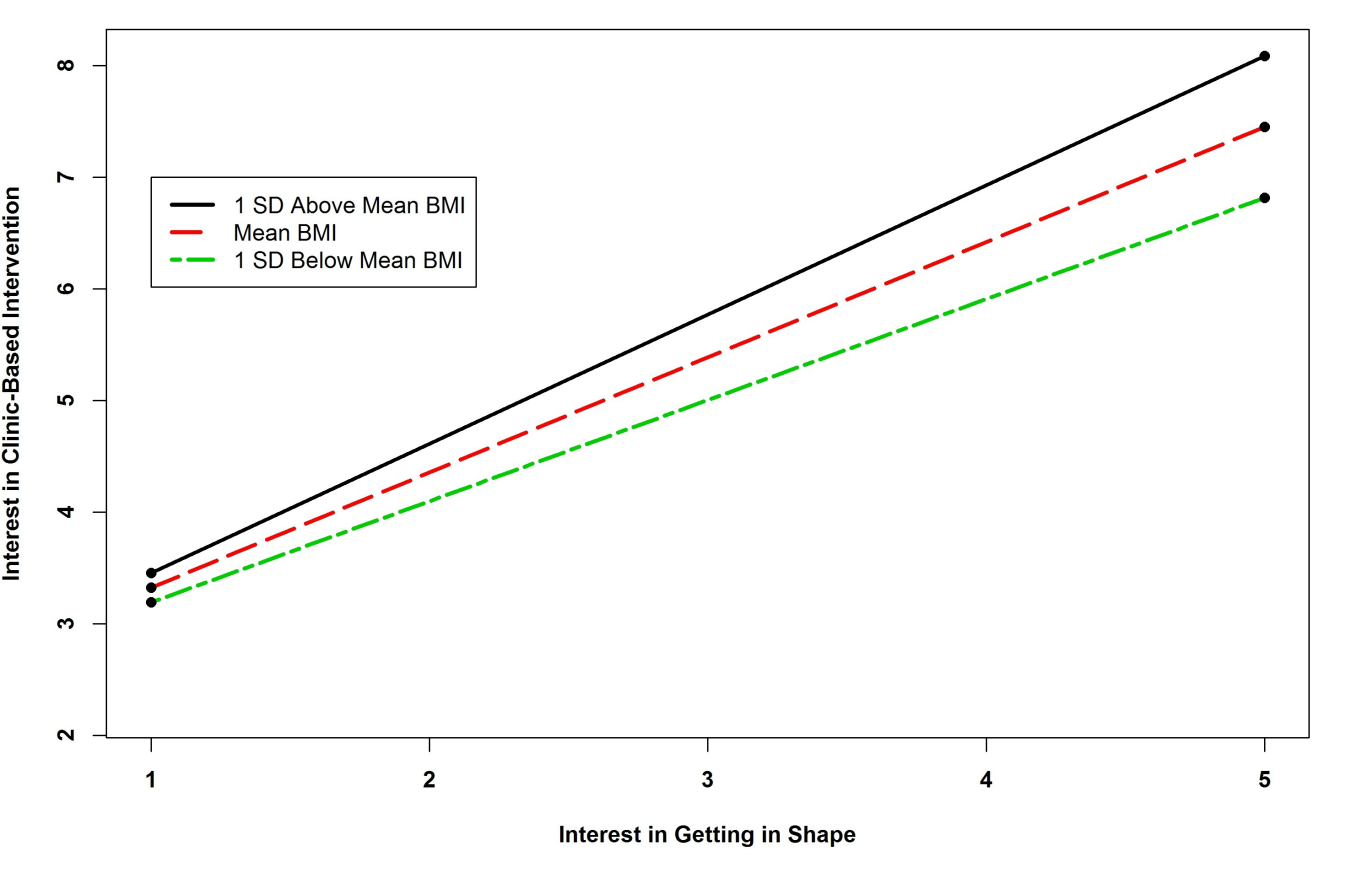
Simple slopes showing relationship between BMI and interest in getting in shape interaction and interest in clinic-based intervention.

Table 3 shows the results for each regression model. The results of the regression analysis of interest in clinic-based interventions indicated that there were two statistically significant predictors: (1) fruit and vegetable consumption and (2) the interaction term between BMI and interest in getting in shape (exercise). Probing this interaction revealed that all simple slopes were positive and significant (*B=*1.158, *P=*.002; *B=*1.032, *P<*.001; and *B=*0.906, *P<*.001, for one standard deviation above the mean BMI, the mean BMI, and one standard deviation below the mean BMI, respectively), indicating that as BMI increased, the relationship between interest in getting in shape and interest in a clinic-based (ie, face-to-face) intervention was stronger. Figure 1 shows this interaction.

**Table 3:** Predictors of each regression of intervention modality and *R* ^2^ in each model.

Predictor		Intervention type, unstandardized beta coefficient (standard error)					
		Clinic	Telephone	Computer		Smartphone	
**Demographics**							
	Age	-0.006 (0.004)	-0.002 (0.004)		-0.004 (0.004)		-0.021 (0.004)^c^
	BMI	-0.001 (0.008)	-0.005 (0.008)		0.006 (0.008)		-0.009 (0.007)
	Sex	0.123 (0.238)	0.106 (0.259)		-0.271 (0.253)		-0.009 (0.211)
	Cancer site	-0.210 (0.124)	-0.124 (0.134)		0.078 (0.131)		0.027 (0.111)
	Education	0.055 (0.048)	-0.036 (0.051)		0.095 (0.050)		0.045 (0.042)
**Technology use and access**							
	Have computer	-0.049 (0.151)	-0.342 (0.167)^a^		0.596 (0.150)^c^		-0.316 (0.139)^a^
	Have access to Internet	-0.045 (0.033)	0.006 (0.032)		-0.018 (0.032)		0.024 (0.032)
	Use social networking sites	0.107 (0.100)	0.047 (0.100)		0.392 (0.100)^c^		0.184 (0.094)^a^
	Use Web camera	0.100 (0.100)	0.050 (0.104)		0.287 (0.097)^b^		0.397 (0.103)^c^
**Behavioral goals**							
	Getting in shape (exercise)	0.395 (0.057)^c^	0.231 (0.063)^c^		0.249 (0.068)		0.092 (0.053)
	Eating better to stay healthy	0.070 (0.051)	0.152 (0.064)^a^		0.334 (0.067)^c^		0.055 (0.054)
	Weight control	0.071 (0.055)	0.151 (0.054)^b^		-0.050 (0.062)		0.089 (0.050)
**Current behavior**							
	Godin score, physical activity (PA)	0.002 (0.002)	0.002 (0.002)		0.003 (0.002)		0.004 (0.002)^a^
	Daily servings of fruits and vegetables (FV)	0.045 (0.019)^a^	0.043 (0.019)^a^		0.054 (0.019)^b^		0.014 (0.019)
	Age × PA	<0.001 (<0.001)	<0.001 (<0.001)		<0.001 (<0.001)		<0.001 (<0.001)
	Age × getting in shape (exercise)	-0.003 (0.005)	0.005 (0.006)		0.001 (0.006)		-0.001 (0.005)
	Age × eating better to stay healthy	-0.007 (0.004)	-0.006 (0.006)		-0.007 (0.004)		-0.010 (0.004)^b^
	Age × weight control	0.005 (0.004)	-0.002 (0.005)		<0.001 (0.004)		0.000 (0.004)
	BMI × getting in shape (exercise)	0.023 (0.011)^a^	0.023 (0.012)^a^		-0.009 (0.013)		0.018 (0.009)^a^
	BMI × eating better to stay healthy	-0.013 (0.011)	-0.003 (0.011)		0.017 (0.013)		-0.006 (0.010)
	BMI × weight control	0.002 (0.009)	-0.002 (0.008)		-0.001 (0.011)		-0.006 (0.009)
	PA × getting in shape (exercise)	-0.005 (0.003)	-0.008 (0.003)^a^		-0.010 (0.004)^b^		<0.001 (0.002)
	PA × eating better to stay healthy	0.001 (0.003)	0.002 (0.003)		0.006 0.004)		-0.002 (0.002)
	PA × weight control	0.004 (0.002)	0.004 (0.003)		0.001 (0.003)		0.004 (0.002)
	FV × getting in shape (exercise)	-0.030 (0.030)	0.051 (0.031)		0.071 (0.033)^a^		-0.012 (0.024)
	FV × eating better to stay healthy	0.025 (0.027)	0.010 (0.030)		-0.008 (0.027)		-0.012 (0.020)
	FV × weight control	0.010 (0.026)	-0.053 (0.027)^a^		-0.040 (0.027)		0.018 (0.020)

^a^ *P*<.05.

^b^ *P*<.01.

^c^ *P*<.001.

**Figure 2:**
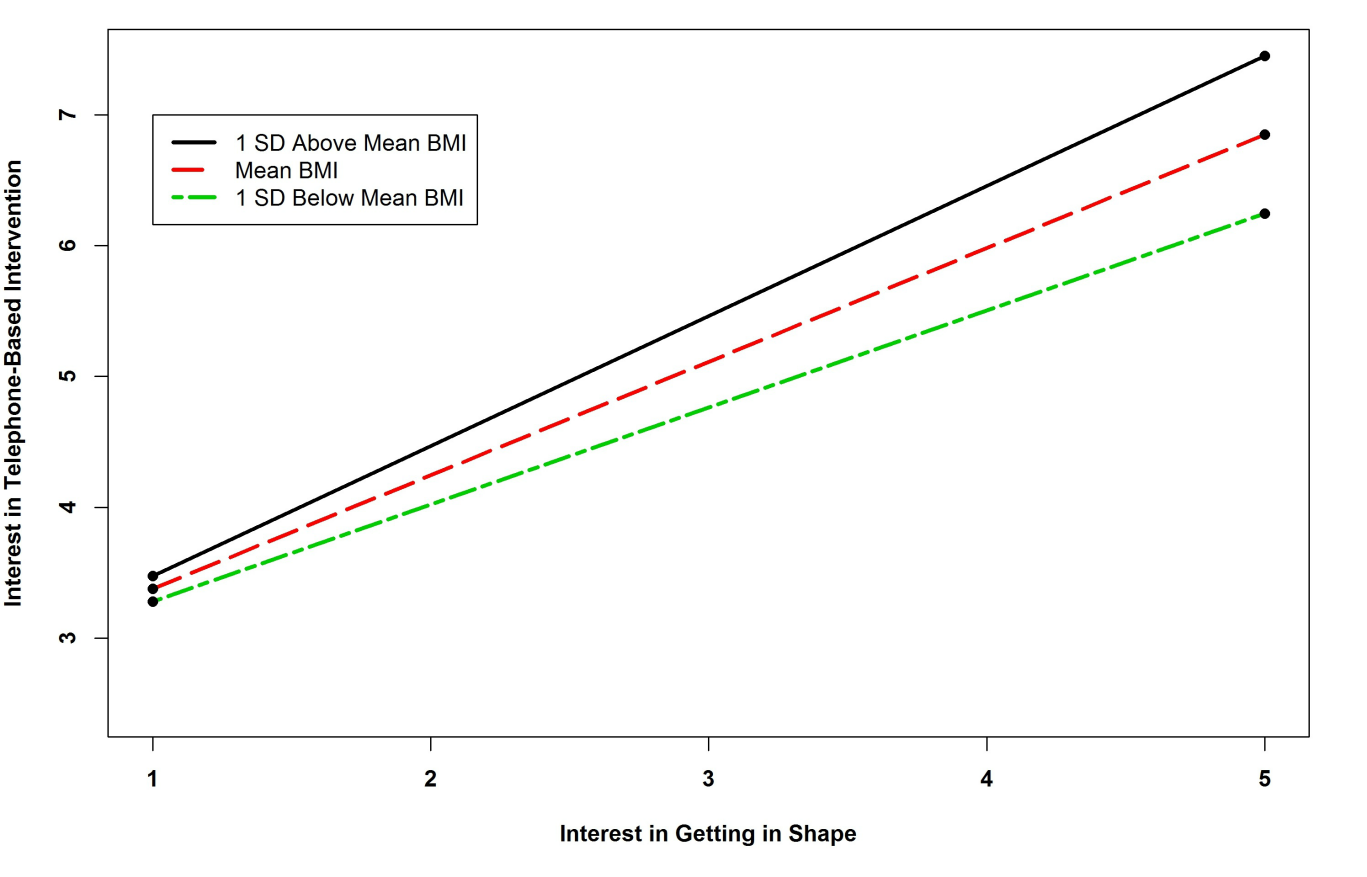
Simple slopes showing relationship between BMI and interest in getting in shape interaction and interest in telephone-based intervention.

**Figure 3:**
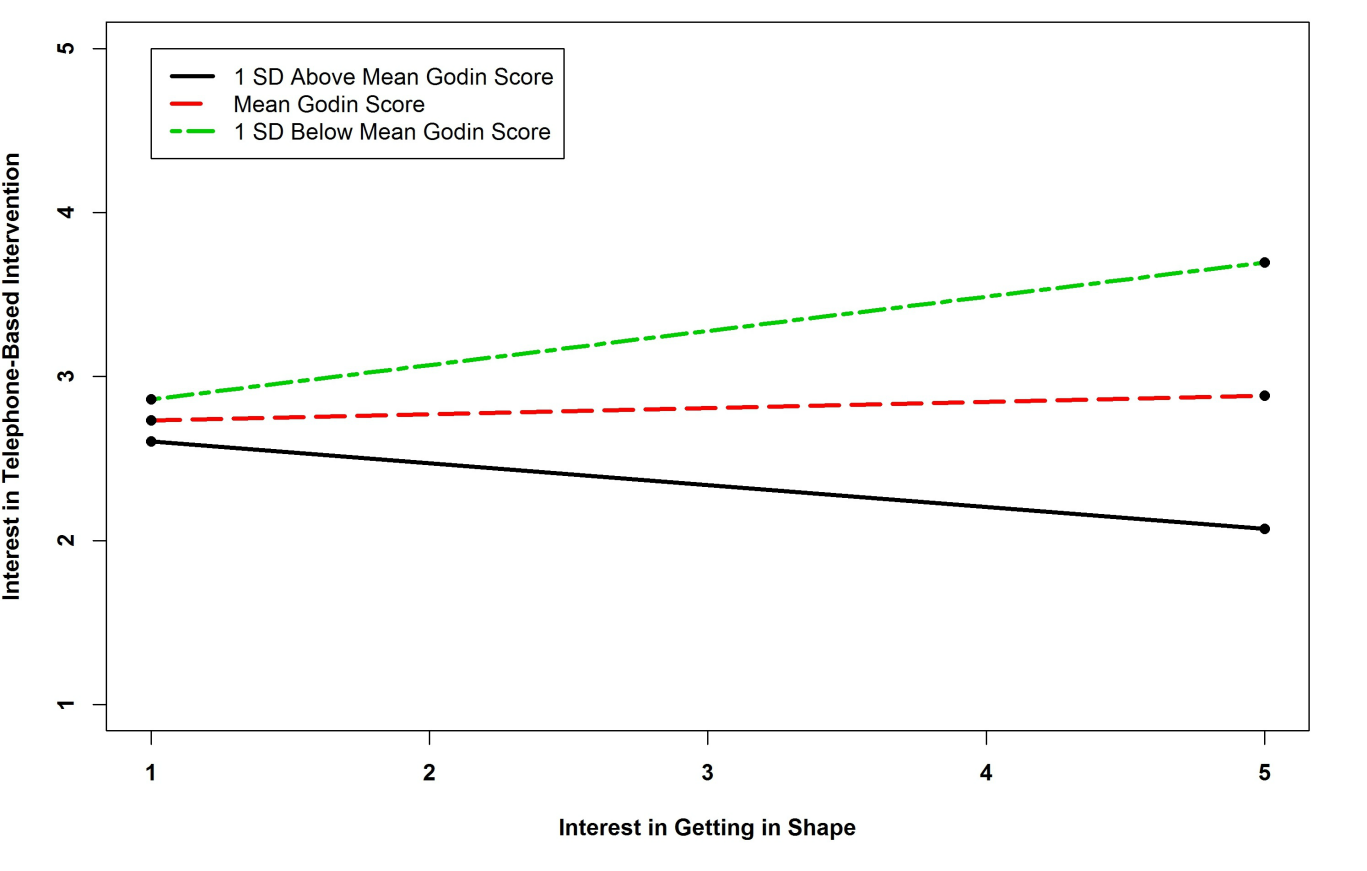
Simple slopes showing relationship between physical activity and interest in getting in shape interaction and interest in telephone-based intervention.

**Figure 4:**
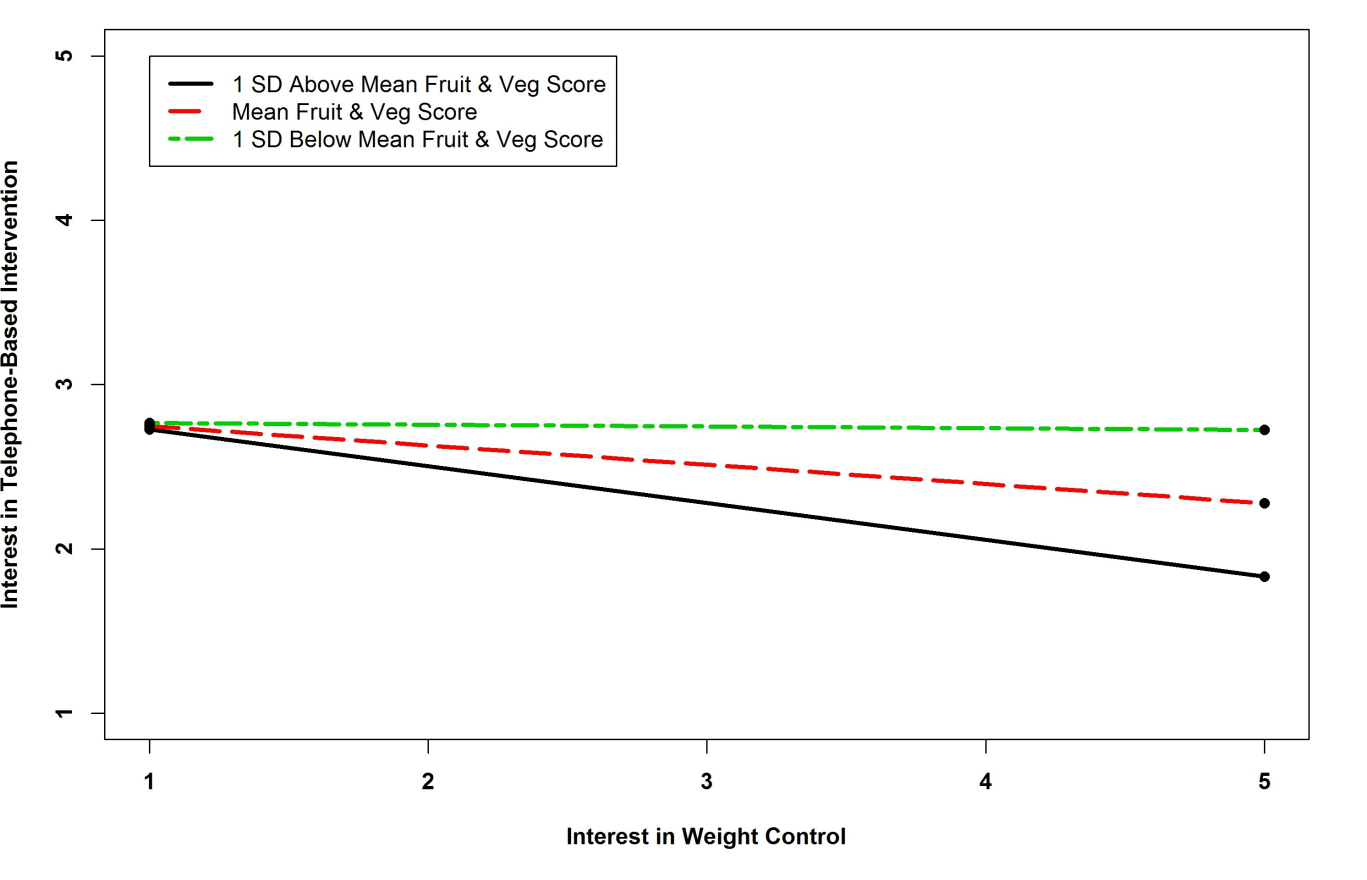
Simple slopes showing relationship between fruit and vegetable consumption and interest in weight control and interest in telephone-based intervention.

The results of the regression of telephone intervention interest showed that there were five statistically significant predictors. Survivors with no computer access were more likely to be interested in telephone-based interventions. Interest in diet interventions was a positive predictor of interest in telephone intervention. The interaction term between BMI and interest in getting in shape and the term between physical activity and interest in getting in shape were significant predictors of interest in telephone-based interventions. The term between BMI and interest in getting in shape was similar to that for clinic-based interventions. Probing the interaction of physical activity by interest in getting in shape revealed that the slope for one standard deviation below the mean Godin Score was positive and significant (*B=*-0.133, *P=*.376; *B=*0.038, *P=*.695; *B=*0.209, *P=*.001), indicating that as physical activity decreased, the relationship between interest in exercise interventions and telephone-based programs became stronger (see Figures 2 and 3). The interaction term between fruit and vegetable consumption and interest in weight management interventions was also a significant predictor of interest in telephone-based interventions. Probing this interaction revealed that none of the simple slopes were significant indicating that the slopes were different from one another but none were significant (*B=*-0.224, *P=*.260; *B=*-0.117, *P=*.425; *B=*-0.011, *P=*.914). See Figure 4.

**Figure 5:**
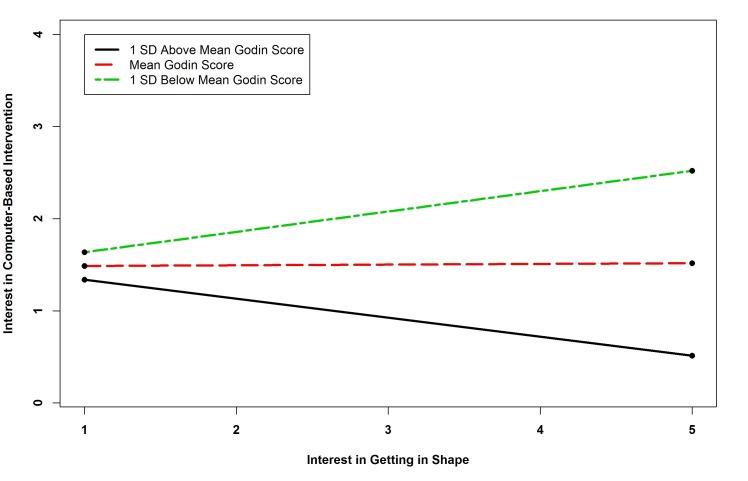
Simple slopes showing relationship between physical activity and interest in getting in shape and interest in computer-based intervention.

**Figure 6:**
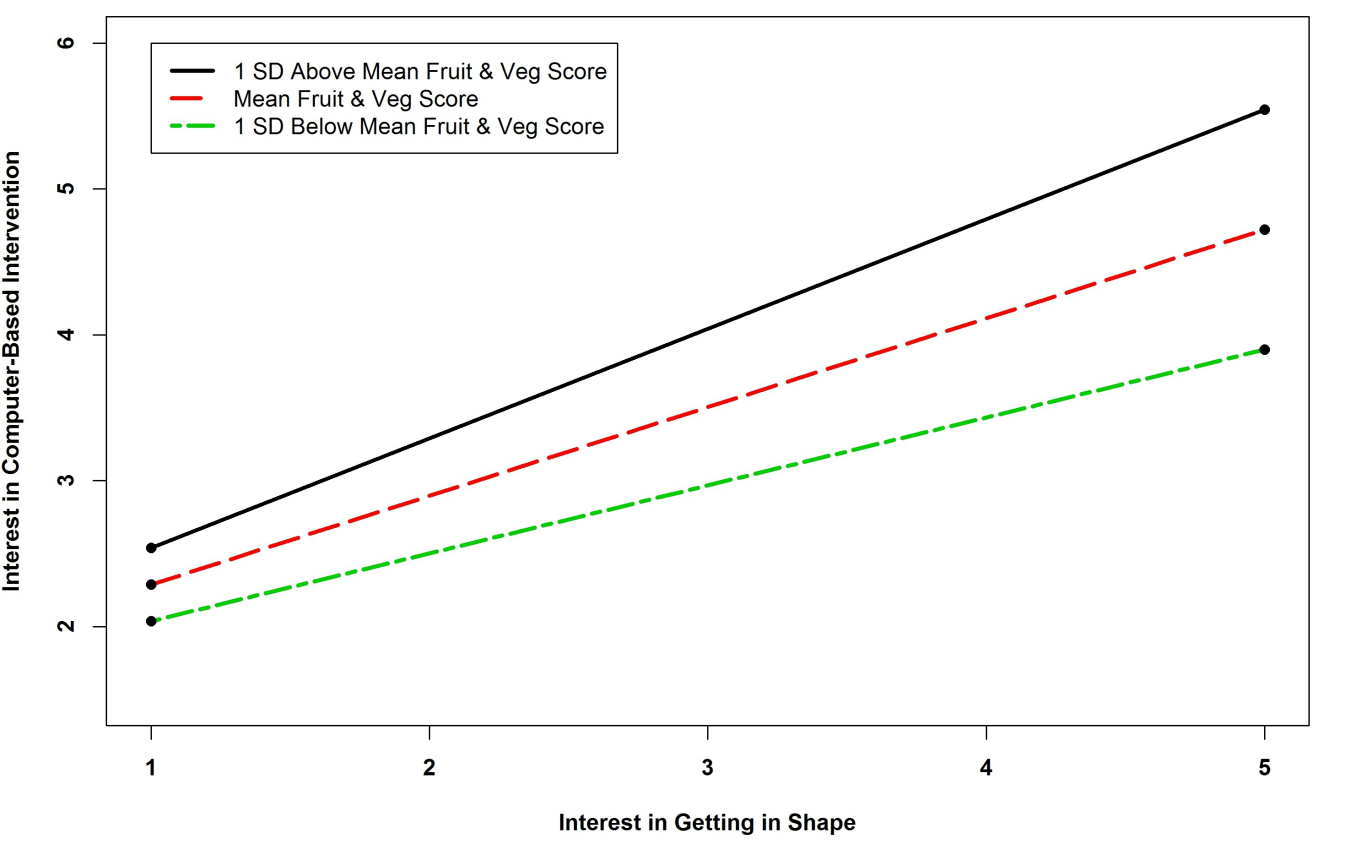
Simple slopes showing relationship between fruit and vegetable consumption and interest in getting in shape interaction and interest in computer-based intervention.

The results of the regression analysis of interest in computer-based interventions revealed six significant predictors. Survivors who had a computer, used social networking sites, and used a Web camera were more likely to be interested in a computer-based intervention. Interest in diet interventions was also a positive predictor. Two significant interaction terms were found. The interaction term between physical activity and interest in getting in shape was negative and significant. Probing the interaction revealed that only the simple slope for one standard deviation below the mean physical activity score was significant (*B*=0.221, *P*=.001), indicating a stronger positive relationship between interest in getting in shape and interest in a computer-based intervention among participants with lower levels of physical activity (see Figure 5). The interaction between fruit and vegetable consumption and interest in getting in shape was also significant. Probing this interaction revealed that all of the slopes were significant and positive (*B=*0.751, *P=*.002; *B=*0.608, *P*<.001; and *B=*0.466, *P*<.001 for one standard deviation above the mean fruit and vegetable consumption score, the mean score, and one standard deviation below the mean score, respectively), indicating that as the number of fruits and vegetables consumed increased, the relationship between interest in getting in shape and interest in a computer-based intervention became stronger (see Figure 6).

**Figure 7:**
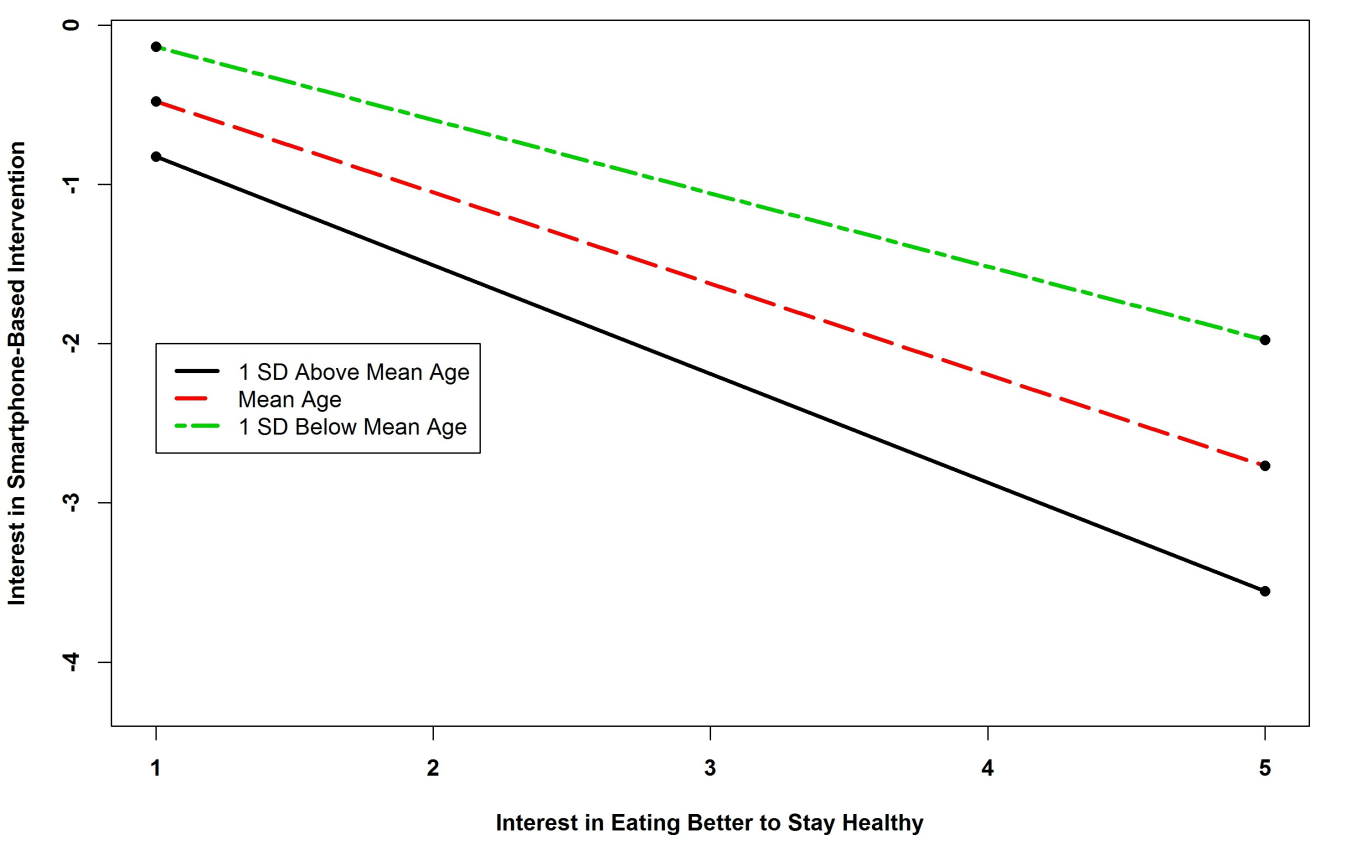
Simple slopes showing relationship between age and interest in healthy eating interaction and interest in smartphone-based intervention.

**Figure 8:**
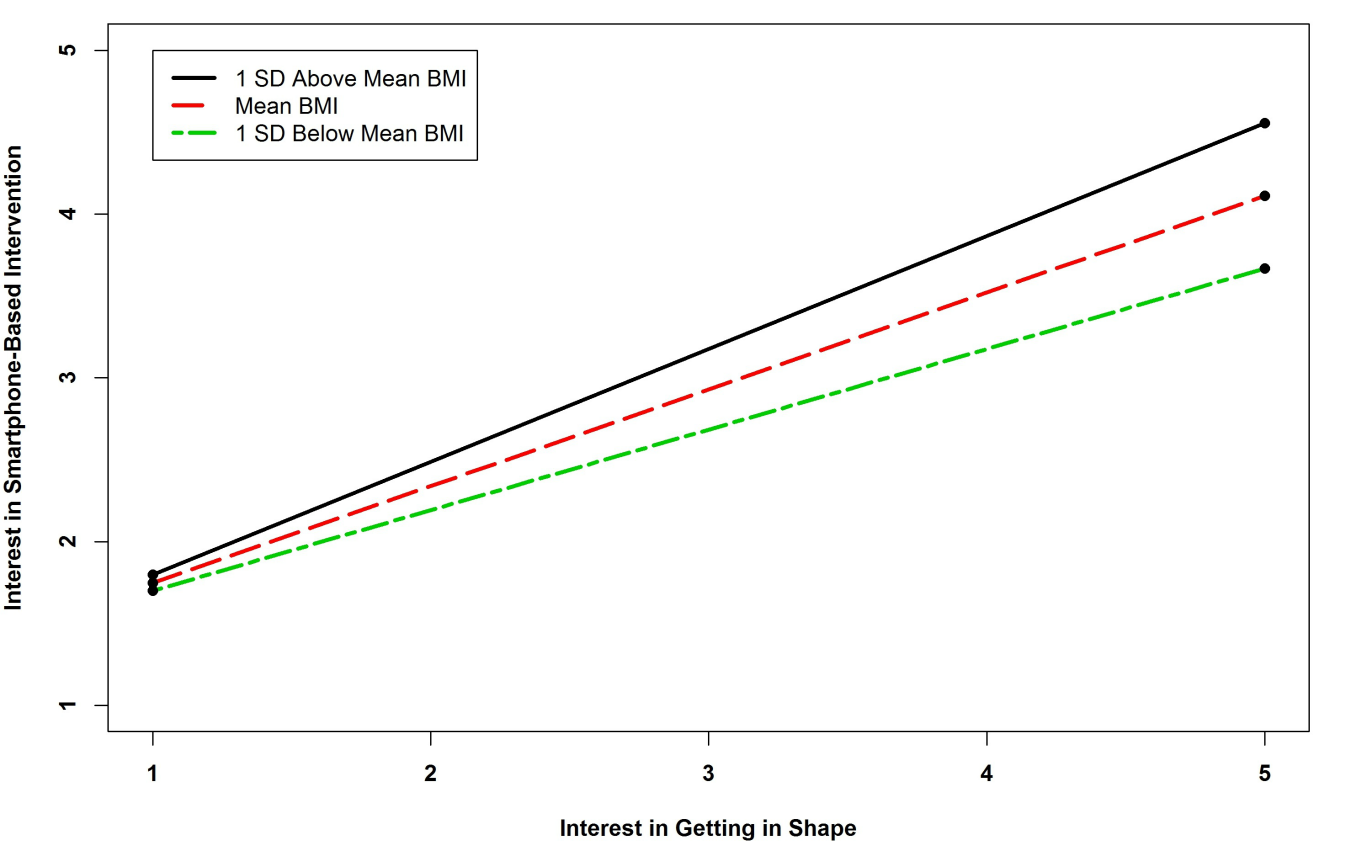
Simple slopes showing relationship between BMI and interest in getting in shape interaction and smartphone-based intervention.

The results of the regression analysis of interest in smartphone interventions revealed six significant predictors. Survivors who did not own a computer, who used social networking sites, and who used a Web camera were more likely to be interested in smartphone-based interventions. Those who engaged in higher levels of physical activity were also more likely to express an interest in this intervention modality. The interaction term between age and interest in healthy eating was significant. Probing this interaction revealed that all simple slopes were negative and significant (*B=*-0.683, *P=*.023; *B=*-0.572 *P=*.026; and *B=*-0.460, *P=*.031 for one standard deviation above the mean age, the mean, and one standard deviation below the mean), indicating that as age increased, the negative relationship between interest in a diet intervention and interest in a smartphone-based intervention became stronger (see Figure 7). The interaction term between BMI and interest in getting in shape was significant. Probing this interaction showed that all simple slopes were positive and significant (*B=*0.689, *P<*.001; *B=*0.5912 *P=*.021; and *B=*0.492, *P=*.018 for one standard deviation above the mean BMI, the mean, and one standard deviation below the mean), indicating that as BMI increased, the relationship between interest in getting in shape and interest in a smartphone intervention became stronger (see Figure 8).

The correlations among the outcome variables are presented in Table 4. All outcome variables were significantly correlated, with the highest correlations being between clinic and telephone intervention interest (*r*=.539) and computer and smartphone intervention interest (*r*=.368).

**Table 4:** Correlations of interest in intervention modalities^a^.

Intervention modality	Clinic	Telephone	Computer	Mobile phone
Clinic	–			
Telephone	.539	–		
Computer	.217	.315	–	
Smartphone	.199	.257	.368	

^a^All *P*s<.001.

## Discussion

### Principal Findings

In this study, we investigated cancer survivors’ use of various forms of technology, as well as their interest in the delivery modality of health behavior change interventions. The purpose was to better understand what factors predict interest in newer forms of technology, such as smartphones, versus more traditional channels of delivery (eg, face-to-face, clinic-based interventions). The results indicate a low level of interest in interventions delivered via smartphone, with nearly 70% of the sample reporting no interest at all in this modality. This finding is expected, given that the average age of survey respondents was over 60 years, which is the age group with the lowest reported rate of ownership of smartphones [36]. In contrast, computer-based programs received the highest rating for “very” to “extremely” interested, indicating a higher level of overall interest in and acceptability of health interventions delivered through this medium. This interest is further supported by the high level of computer ownership in the sample (88.7%) and access to the Internet in the home (62.3%). Despite these high levels of preference for and ownership of computers, a relatively low number of participants reported using Internet-based social networking sites (30.3%) or Web cameras (23.6%), indicating that interventions using these technologies may reach only a small portion of cancer survivors who have a computer and access to the Internet.

Several predictors in the model were shown to be significant in predicting interest in technology-based interventions. Use of other technology-based platforms, such as social networking sites and Web cameras, positively predicted interest in interventions using broad-reaching technologies, such as computers and smartphones. Participants who did not have a computer were more likely to be interested in smartphone interventions. These individuals are part of a growing “smartphone-dependent” population in America that tends to be of low income and educational attainment [37]. In addition, participant age played a role in a significant interaction term for this modality, indicating that for some types of behavioral interventions (ie, diet) smartphone delivery may be of less interest to older survivors. When targeting cancer survivors for intervention, it is important that current technology use and age be taken into consideration. Some cancer survivors may be more amenable to the use of smartphones for interventions, such as those who already use various technology platforms or are younger.

We explored 13 interaction terms in this study. Of these, eight showed significant moderation. No one health behavior or demographic moderated the relationship between intervention type and modality alone. This highlights the complexity of the relationship between survivor interest in intervention modality, behavioral intervention targets, and current health behaviors. For example, the relationships between interest in getting into shape and clinic-based, telephone-based, and smartphone-based interventions were positive and significant across all BMI categories, though this interaction was not significant for computer-based interventions, which had received the highest overall interest rating. This demonstrates that although generally computers may be a modality of high interest for health behavior intervention in this population, when specifically looking at exercise interventions, other broad-reaching modalities may be a better fit for both reach and retention. It should be noted that this relationship was strongest for those who were obese, suggesting this is particularly relevant for those with a high need for this type of behavioral intervention. Additionally, individuals with lower physical activity levels reported a positive relationship between interest in getting in shape and interest in computer-based interventions. However, this relationship was negative for individuals with higher levels of physical activity. This information would be clinically relevant when recommending a computer-based exercise intervention to a survivor, such that individuals with low levels of physical activity may show stronger preference towards a computer-based exercise intervention, while those with higher levels may prefer the intervention delivered by a different modality. In this way, current health behaviors, as with current technology use, are also important factors to consider when targeting cancer survivors for intervention. It should be noted that these findings are independent of computer ownership.

These findings support those of previous research regarding health behavior change interventions among cancer survivors and these survivors’ interest in traditional versus technologically mediated channels of delivery. In their formative research for a mobile-enabled Web app to promote physical activity in older cancer survivors, Hong et al [27,28] found that while participants were enthusiastic regarding participation in an online health intervention, less than 10% reported accessing the Internet through their smartphones. In a design survey, 80% of 92 interview participants [27] reported that they would participate in an online physical activity program, but only 56% of pilot participants indicated that they would continue using the program after the intervention had completed [28]. In addition, participants typically accessed the app via a desktop or laptop computer, with only 9% accessing it through a smartphone [28]. Our findings support the results of this formative work, indicating that overall interest in health behavior interventions is low in this population and Web apps accessed via the computer may be preferable to those accessed via smartphone.

### Strengths and Limitations

There were several strengths of this study. Not only was interest in multiple intervention modalities evaluated in this at-risk population, but specific behavioral predictors of this interest were also evaluated, allowing the intervention modality to be better targeted in the future. Evaluating the relationships that exist between interest in health behavior interventions and the delivery modality allows for greater specificity, instead of defaulting to a “one size fits all” approach for intervention delivery. These data allow us to analyze which types of interventions are better suited for delivery by a particular modality, and to whom. In addition, the large sample size facilitated the use of statistical methods that were amply powered to detect these relationships.

A limitation of this study was that the cross-sectional design only allowed participants to indicate an interest in various types of interventions, rather than actual participation. Therefore, predictor variables are only suggestive of having predictive value in future studies. Although survivors may have reported high interest levels, a longitudinal follow-up study is needed to determine whether this interest translates into actual engagement. Also, participants were able to rate their interest in each modality separately; thus, preferences between modalities could not be fully assessed. In the future, asking patients to rank order their preference between modalities would facilitate a better understanding of their “top choice” for intervention delivery. In addition, while most individuals who received the mailed survey responded, the percentage of those who did not respond may have biased our data. Finally, the data were collected in 2010. Since this time, smartphone use in the general population has nearly doubled. However, this is not the case for older adults in America—who are the focus of this paper—who have had a much slower trajectory of smartphone uptake [37].

### Conclusions

These results provide a better understanding of the individual factors that predict acceptance of health behavior intervention modalities among cancer survivors. Research has found substantial support for the efficacy of broad-reaching channels of delivery for health behavior intervention with cancer survivors [38]. As this population is growing, it is important to consider not only the most effective way to reach these individuals but also the most efficient and acceptable method of providing health behavior interventions. Future research in health behavior change intervention among cancer survivors should take into account multiple factors when choosing the channel of delivery for intervention, including age, experience and comfort with technology, and health behavior and conditions. Given the delayed adoption of technology among cancer survivors who tend to be older, it is likely that the use of smartphone-based interventions may be more acceptable in the coming years.

## Abbreviations

BMI: Body Mass Index
FV: daily servings of fruits and vegetables
PA: physical activity
